# The genetics of urinary microbiome, an exploration of the trigger in calcium oxalate stone

**DOI:** 10.3389/fgene.2023.1260278

**Published:** 2023-10-03

**Authors:** Yuanyuan Yang, Lintao Miao, Yuchao Lu, Shaogang Wang

**Affiliations:** Department of Urology, Tongji Hospital, Tongji Medical College, Huazhong University of Science and Technology, Wuhan, Hubei, China

**Keywords:** CaOx stones, renal pelvis urine, microbiomics, 16S rRNA gene sequencing, genetics

## Abstract

**Background:** Kidney stone disease is a global disease; however, it has not been totally understood. Calcium oxalate (CaOx) stone is the dominant type of kidney stone, and the potential factors involved in its formation are yet to be explored. Clinically, we found that the CaOx stones in patients were mainly unilateral; therefore, systemic factors cannot explain them, although some local factors must be involved. Urinary microbiota is involved in stone formation. Therefore, this study aimed to explore the association between the urinary microbiota and CaOx stones and provide insight into the medical treatment and prevention of CaOx stones.

**Methods:** Sixteen pelvic urine samples were collected from the stone and non-stone sides of patients with unilateral CaOx stones following strict criteria. The 16S rRNA gene sequencing was performed on each pair of pelvic urine samples at the species level. Many bioinformatic analyses were conducted to explore the potential factors affecting CaOx stone formation.

**Results:** Although no statistically significant difference was found between the overall microbiota of the pelvis urine from the two sides. Many biologically distinct taxa were observed, including many bacteria found in previous studies, like Proteobacteria, Actinobacteria, Firmicute and *Enterobacter cloacae* and so on. What’s more, despite these common bacteria, our current study added to these bacterial communities with additional identification of Deinococcus-Thermus, Coriobacteriia, Porphyromonas and Ralstonia. To predict the functions of these microbiota, Kyoto Encyclopedia for Genes and Genomes and MetaCyc analysis were conducted and immunometabolism might be an important pathway. Moreover, a random forest predictor was constructed to distinguish the stone side from the non-stone side, with an accuracy of 62.5%.

**Conclusion:** Our research profiled the microbiome in the pelvis urine from both the stone and non-stone sides of patients with unilateral CaOx stones, provided insight into the dominant role of urinary dysbiosis in CaOx stones formation. Furthermore, this study also predicted the potential crosstalk among urinary microbiota, immunometabolism, and CaOx stones.

## 1 Introduction

Kidney stone disease is a global disease with high morbidity and recurrence rates that result in significant health and economic burdens on both individuals and society (Sorokin et al., 2017). There are approximately 5.8% of patients with kidney stones in China, of whom 6.5% and 5.1% are males and females, respectively (Zeng et al., 2017). Although the surgical treatment of kidney stones has improved significantly, the medical treatment and prevention of kidney stones remain stagnant because the underlying mechanism is still unclear (Zisman, 2017). There are many types of kidney stones, of which calcium oxalate (CaOx) stone is predominant, with an occurrence rate of 80% (Howles and Thakker, 2020). However, the pathogenesis of CaOx stones is complex and remains unclear. Accumulating evidence has implicated genetics, infection, immune disorders, and metabolites to be closely related to CaOx stones (Okada et al., 2009; Taguchi et al., 2017). Taguchi et al. demonstrated that patients with CaOx stones showed upregulation and downregulation of genes associated with the M1 phenotype polarization of macrophages and genes associated with M2 phenotype polarization, respectively (Taguchi et al., 2016). Over the last few decades, the role of microbiomes has drawn considerable attention worldwide. A previous study found that urinary microbiota is closely related to infectious stones such as struvite stones via urease (Espinosa-Ortiz et al., 2019). However, with the rapid development of 16S rRNA gene sequencing, an increasing relationship between microbiota and metabolic stones, such as CaOx stones, has been revealed. Dornbier et al. (2020) reported that several taxa were enriched in stones compared to the surrounding urine, indicating that microbiota might play important roles in CaOx stone formation. Barr-Barr-Beare et al. (2015) reported that E. coli contributed to the formation of CaOx stones. Additionally, microbiota-induced immunometabolism has been found to be important in many diseases (Dong et al., 2023; Samami et al., 2023), and Xi et al. (2019) proposed that short-chain fatty acids (SCFAs) produced by the gut microbiota are associated with CaOx stones. Fachi et al. (2020) reported that *Clostridium difficile* can modulate neutrophils and innate lymphoid cells via acetate. Given the plethora of proposed mechanisms, there is an urgent need to understand the immune-based mechanisms underlying CaOx stone formation. Furthermore, understanding the crosstalk among immunometabolism, microbiota, and kidneys will help explore the mechanism and provide insight into the novel medical treatments or prevention of CaOx stones. Therefore, to further explore this relationship, we conducted 16S rRNA sequencing of the pelvic urine samples of patients with unilateral CaOx stones to determine the occurrence of almost unilateral CaOx stones. Moreover, previous studies frequently collected bladder urine samples, which could not accurately reflect the environment of the stone side; Though Dornbier et al. reported there were no significant difference between the bladder urine and paired upper tract urine, they did not compare the stone side and non-stone side pelvis urine of the same unilateral stone patients. Therefore, we collected the pelvis urine samples from the stone and non-stone sides as a self-control. This study may provide insight into the mechanisms of and potential medical treatments for CaOx stone formation.

## 2 Materials and methods

### 2.1 Recruitment of patients and samples collection

We established strict inclusion and exclusion criteria to rule out additional factors that affect the microbiota of pelvis urine. Patients suspected of having unilateral CaOx stones on computed tomography were enrolled in the study. Individuals with urinary tract infections (UTIs), other urological diseases, or those treated with antibiotics within the past 4 weeks were excluded. Finally, we collected 16 pelvis urine samples from 8 unilateral stone formers at Tongji Hospital. All patients underwent percutaneous nephrolithotomy, and the stones removed during surgery were sent for compositional analysis. Pelvis urine samples from both the stone and non-stone sides were collected preoperatively. Furosemide was intravenously injected to accelerate the discharge of pelvis urine samples, which were subsequently collected from the two sides in asepsis tubes through a ureteral catheter. The entire sample collection process, subsequent DNA extraction, and library preparation were conducted under the guidance of MICROCOSM (an international consortium for the microbiome in urinary stone disease) to minimize technical biases and barriers (Kachroo et al., 2021). This study was approved by the Ethical Review Board of Tongji Hospital, Tongji Medical College, and Huazhong University of Science and Technology (2021S130). Informed consent was obtained from all participants.

### 2.2 16S RNA gene sequencing and processing

The pelvis urine obtained from both two sides were at least 3mL, after collected, the urine samples were immediately transferred to the lab without adding any preservation in liquid nitrogen. The whole procedure was within 15 min and the samples were transferred in liquid nitrogen. Total DNAs were extracted using the OMEGA Soil DNA Kit (D5625-01) (Omega Bio-Tek, Norcross, GA, USA) and stored at −20°C for further analysis. Paired-end sequencing of the community DNA fragments was performed using the Illumina platform. Detailed information of the specific sequencing process could be found in our previous study (Yang et al., 2022). Then the raw sequences were analysed with QIIME2. Each deduplicated sequence generated after the use of DADA2 quality control was termed an amplicon sequence variant (ASV) or a characteristic sequence, and the abundance table of these sequences in the sample was known as a characteristic table (corresponding to the OTU table). The method of denoising and generating feature sequences represented by DADA2 was developed using a mainstream analysis platform (QIIME2). DADA2 was not adapted to all amplicons, we still retained the Vsearch method based on OTU clustering as an alternative. First, cutadapt was used to remove the primer fragment of the sequence, then discarded the sequence without matching primers. Concatenated sequences using Vsearch’s fastq_mergepairs module. Fastq_filter module was used to control the quality of the splicing sequence. The derep-fulllength module was used to remove repetitive sequences. The cluster size module was used to cluster the deduplicated sequences at the 98% similarity level, and the uchime_*denovo* module was used to remove chimeras. Then used the cluster_size module, high-quality sequences were clustered at the 97% similarity level and representative sequences and OTU tables were output, respectively. Finally, singletons OTUs (that is, otus with an abundance of 1 in all samples, the default operation) and their representative sequences were removed from the OTU table. We then used the RDP FrameBot software (https://github.com/rdpstaff/Framebot), which was based on the corresponding functional genes of the RDP website, to download the seeds of protein sequences and the nucleic acid sequence of insertion and deletion errors to correct. The amino acid length filtering threshold was set to 50; the *de novo* mode was used to add the validated protein sequences that fulfilled the specific requirements for the reference sequence, while the default values were used for other parameters. After FrameBot analysis, the corrected nucleic acid and protein sequences were available, whereas the non-target fragment was removed, and the corrected nucleic acid sequence was used for subsequent analysis.

### 2.3 Taxonomic annotation of species and flattening

The essence of species annotation is the process of comparison with a reference sequence database and scoring of the results. Therefore, database selection is crucial. A good species annotation database should cover all species of sequences to be tested and reduce other non-tested species as much as possible. Consequently, it can improve true positives and reduce false positives, ultimately leading to an improvement in species annotation resolution. The QIIME2 classify-sklearn algorithm (Bokulich et al., 2018) (https://github.com/QIIME2/q2-feature-classifier) was used for the taxonomic annotation. Species annotation was performed for the characteristic sequences of each ASV or representative sequences of each OTU using the pre-trained Naive Bayes classifier with default parameters in the QIIME2 software. The abundance table of ASV/OTU was generated in the previous analysis step, and the subsequent part of the analysis step required each sample to be performed at the same sequencing depth level; therefore, the table had to be transformed to some extent. Furthermore, a rarefaction method can also be used to achieve a uniform depth by randomly extracting a certain number of sequences from each sample.

### 2.4 Microbial diversity analysis and identification of differential taxa

Based on the taxonomic abundance profiles, a table showing the specific compositions of the microbial communities in each sample at each taxonomic level was obtained. From this table, the number of taxa in different samples at each taxonomic level was calculated. Alpha diversity is known as within-habitat diversity. Richness and evenness are measures of alpha diversity. Chao1 and Observed Species are two measures of richness. Pielou represents the envenness. Shannon is a composite that accounts for both richness and evenness. Simpson and Faith’s PD indicate the diversity based on evolutionary diversity. Good’s coverage characterizes the coverage of alpha diversity. The methods for calculating these alpha diversity indices are found at http://scikit-bio.org/docs/latest/generated/skbio.diversity.alpha.html#module-skbio.diversity.alpha. Beta diversity refers to the difference in species composition or the rate of species replacement along environmental gradients among different communities, and is also known as between-habitat diversity. Multi-dimensional microbial data can be reduced using principal coordinate analysis (PCoA), PCoA analysis was performed on the distance matrices. A Venn diagram was constructed to determine the common and unique species among the different groups. Heat maps were used for species composition analysis to further compare the differences in species composition between the samples and show the species abundance distribution trend in each sample. Linear discriminant analysis (LDA) Effect Size (LEfSe) combined the nonparametric Kruskal–Wallis test with LDA. LEfSe was conducted to find robust biomarkers between groups. Random forest is a classical and efficient machine learning algorithm based on a decision tree. It was conducted to deeply explore the complex non-linear interdependence between variables. This particularly applies to microbial community data frequently presenting discrete and discontinuous distributions. Random forest analysis was performed by calling the “classify_samples_ncv” function in the q2-sample-classifier to identify the marker species. Phylogenetic Investigation of Communities by Reconstruction of Unobserved States (PICRUSt2) was used to predict the functional abundance of a sample based on the sequence abundance of marker genes in the sample (Gavin M. Douglas, et al., P. preprint), it was just a prediction and discussion of possible functions, it could not lead to clear and assured results. The16S rRNA gene sequences were predicted in the MetaCyc (https://metacyc.org/) and Kyoto Encyclopedia for Genes and Genomes (KEGG) (https://www.kegg.jp/) databases using the PICRUSt2.

## 3 Results

### 3.1 Detailed information of the enrolled patients and 16S rRNA gene sequencing

**FIGURE 1:**
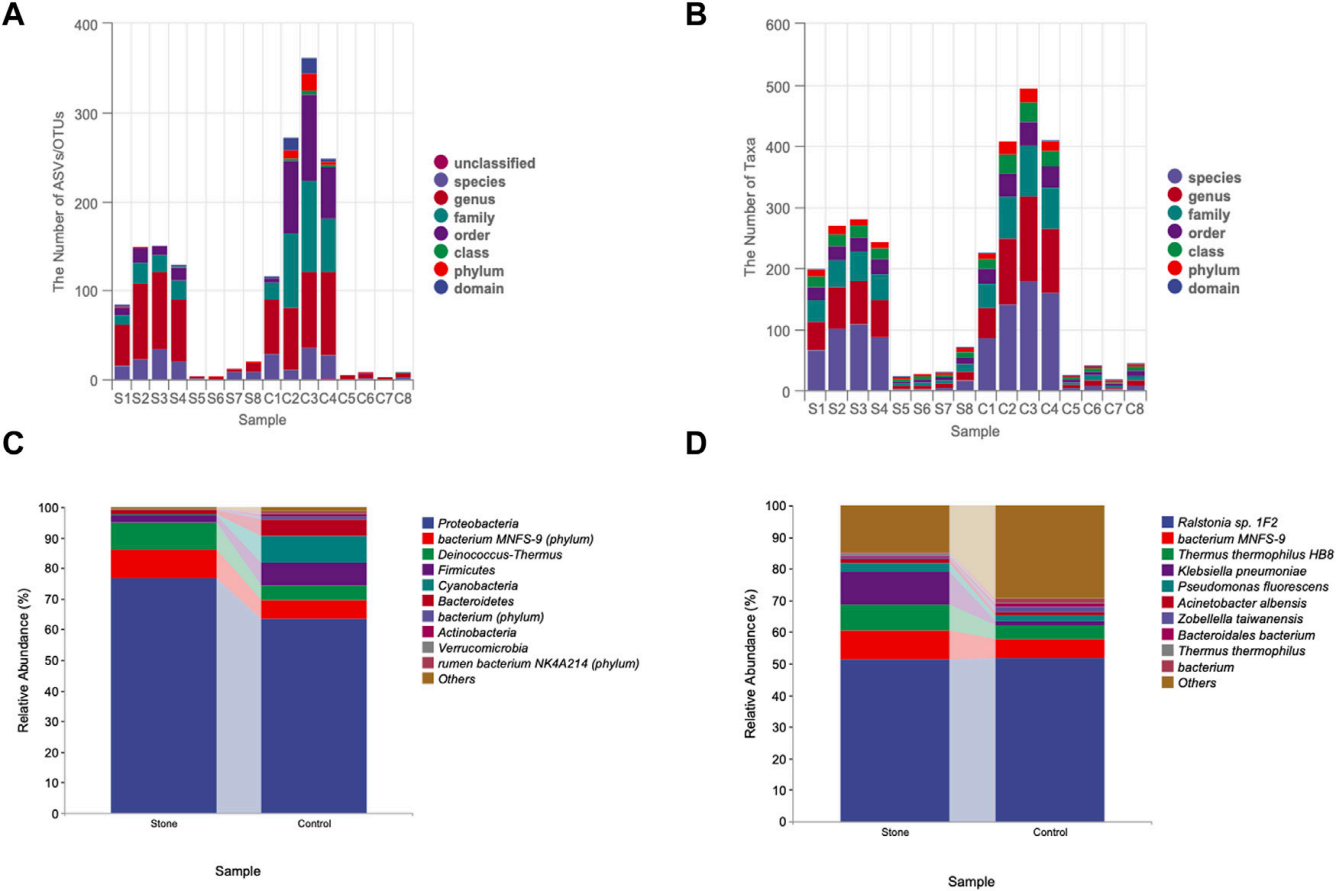
Quality control of 16S rRNA sequencing. **(A)** Annotation accuracy of the samples. The abscissa represents the number ASV/OTU of each sample, and the ordinate represents the highest annotated classification level of domain, phylum, class, order, family, genus, and species in the annotation results. Different levels were identified by different colors, and the column heights correspond to the number of ASV/OTUs. **(B)** Taxonomic unit count. The ordinate represents the number of taxa up to the species level. Different taxonomic levels were identified by different colors, and the height of the column indicates the number of taxa. The difference composition of the urine samples intergroup at the phylum **(C),(D)** species levels. ASV, amplicon sequence variant; OTU, operational taxonomic unit.

Patients with unilateral CaOx stones were enrolled. No antibiotics were administered within 1 week before the pelvic urine sample collection. Pelvic urine samples were collected from both the stone and non-stone sides of the enrolled patients during percutaneous nephrolithotomy. In total, we collected 16 samples: 8 each from the stone and non-stone sides. Table 1 presents the detailed information of the eight participants. The patients comprised 6 males and 2 females with a mean age of 52. Subsequently, 16S rRNA gene sequencing was conducted on the pelvic urine samples of the patients. 91503 ASVs were found in 16 samples, with an average of 5719 in each sample. Additionally, the DADA2 method was not used for all amplicons; therefore, we retained the Vsearch method based on OTU clustering. The sequences obtained after processing were compared with reference sequences for species annotation. Annotation accuracy of each sample were presented in Figure 1A, most of the samples could be annotated to species level, however, S6, C5 and C7 were only annotated to genus level (Figure 1A). The specific composition tables of the microbial communities in each sample at each taxonomic level were obtained by flattening the ASV/OTU tables (Supplementary Table S1). The number of taxa present in the different samples at each taxonomic level was calculated using this table (Figure 1B). The number of taxa at species level were the highest in S1, S2, S3, S4, S8, C1, C2, C3, and C4. The composition taxa of the intergroup at phylum level and species level were presented in Figures 1C, D. We could find that Proteobacteria predominated both groups at the phylum level, followed by the bacterium MNFS-9, Deinococcus-Thermus, Firmicutes, Cyanobacteria, and Bacteroidetes. Actinobacteria, Verrucomicrobia, and the rumen bacterium NK4A214 were identified only on the non-stone sides. Furthermore, at the species level, the species on the stone and non-stone sides were similar, and *Ralstonia* sp.1F2, the bacterium MNFS-9, *Thermus thermophius* HB8, *Klebsiella pneumoniae*, and *Pseudomonas fluorescens* were the top five most abundant taxa in both groups, we presumed that there might be no significantly different taxa intergroup (Figures 1C, D). In addition, we conducted analysis of microbiome composition on male and female, separately, and found there were almost no difference between the top10 bacteria in male or female patients (Supplementary Figure S1), previous studies have found differences in the urinary microbiota composition of calcium oxalate stone patients of different genders. However, the urine samples in those previous studies were obtained from bladder urine rather than renal pelvis urine. Additionally, the sample size in our study is not sufficient to support subgroup analysis. Therefore, after expanding the sample size in the future, we will conduct subgroup analysis based on gender once again.

**TABLE 1:** Demographic and clinical characteristics of the enrolled patients.

ID	Age(y)	Gender	BMI (kg/m^2^)	Plasma Ca (mmol/L)	Stone side	Stone type
1	68	Female	23.8	2.31	Right	CaOx
2	42	Male	30	2.25	Left	CaOx
3	42	Male	27.9	2.33	Left	CaOx
4	60	Male	26.3	2.2	Right	CaOx
5	58	Male	25.7	2.45	Left	CaOx
6	48	Female	28.3	2.76	Left	CaOX
7	56	Male	27.5	2.23	Right	CaOx
8	43	Male	25.3	2.32	Left	CaOx

### 3.2 Sequence-based characterization

**FIGURE 2:**
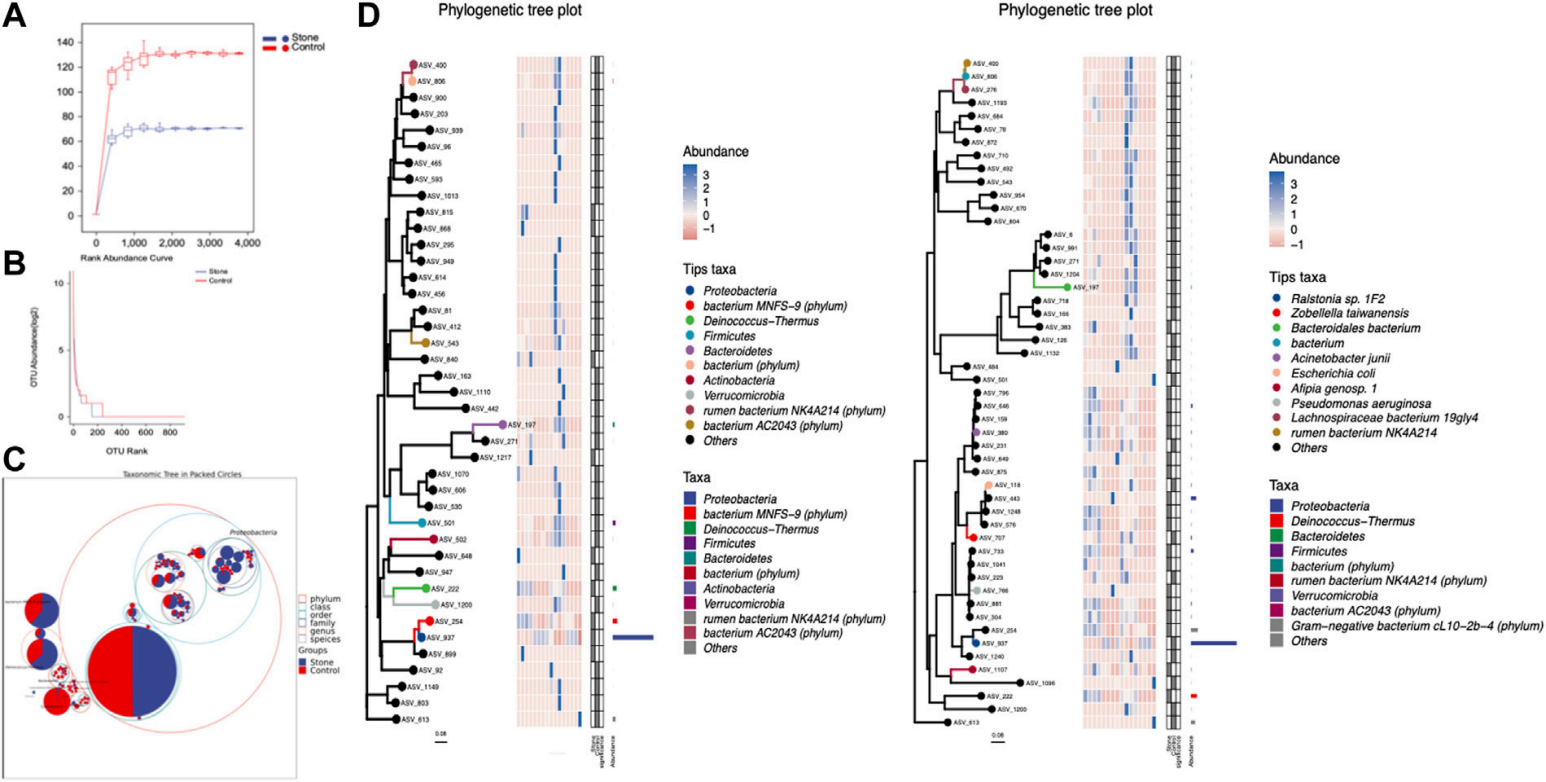
The sequencing profile for the pelvic urine samples from the stone and non-stone sides. **(A)** The gentle rarefaction curve indicates that the sequencing sufficiently reflected the diversity in the pelvic urine samples. **(B)** The flatness of the rank abundance curve reflects the evenness of the community. The evenness of the pelvis urine samples on the non-stone side was higher than that on the stone side. **(C)** Taxonomic tree in packed circles. The largest circle represents the phylum level, and the smaller represents class, order, family, genus, and species. The innermost dot represents the top 100 abundant ASV/OTUs, and the area of dots represents the abundance of ASV/OTUs. The dot is displayed as a pie chart, showing the constituent proportion of these ASV/OTUs in each group. Groups A and B represent the calcium oxalate stone and non-stone sides, respectively. **(D)** Phylogenic tree plot. The phylogenic tree plot, indicating the total abundance of each item at length, is composed of four parts as follows: 1) phylogenic tree;2) abundance heatmap; 3) significantly different heatmap; and 4) bar chart. ASV, amplicon sequence variant; OTU, operational taxonomic unit.

The rarefaction curve is a common analysis, and the total number of species in the samples and the species’ relative abundance can be predicted by randomly sampling a certain number of sequences from the samples. The curve’s flatness indicates that the sequencing depth was sufficient to reflect the diversity of the samples. We found that the control side had higher diversity than stone-side at the same sequencing depth (Figure 2A). In contrast, the flatness of the rank abundance curve reflects the evenness of community composition. The steeper the curve, the lower the evenness of the groups. The eveness of control side was higher than stone side, indicating the abundance of taxa were more similar in control side (Figure 2B). The circle packing chart shows the taxon composition of the microbiota in the pelvic urine samples but indicates the proportion of specific taxa in different groups when combined with pie charts. Particularly, the relative abundance of Proteobacteria in pelvic urine samples from the stone side were higher than those from the non-stone side. The abundance of the bacterium MNFS-9 was also higher on the stone side, whereas Cyanobacteria and Firmicutes were of higher presence on the non-stone side (Figure 2C). The distance of the evolutionary relationships between different species is shown in the vertical direction of the evolutionary tree. The closer they are to each other, the shorter the evolutionary divergence time and the closer the genetic relationship between the two species. The rumen bacterium NK4A214 was closer to bacterium, and Proteobacteria was closer to the bacterium MNFS-9 at the phylum level. However, the rumen bacterium NK4A214, bacterium, and the Lachnospiraceae bacterium 19gly4 appeared to have the same origin at the species level. Proteobacteria was the most abundant among all taxa in terms of phyla and species (Figure 2D).

### 3.3 Biodiversity of the urine microbiome

**FIGURE 3:**
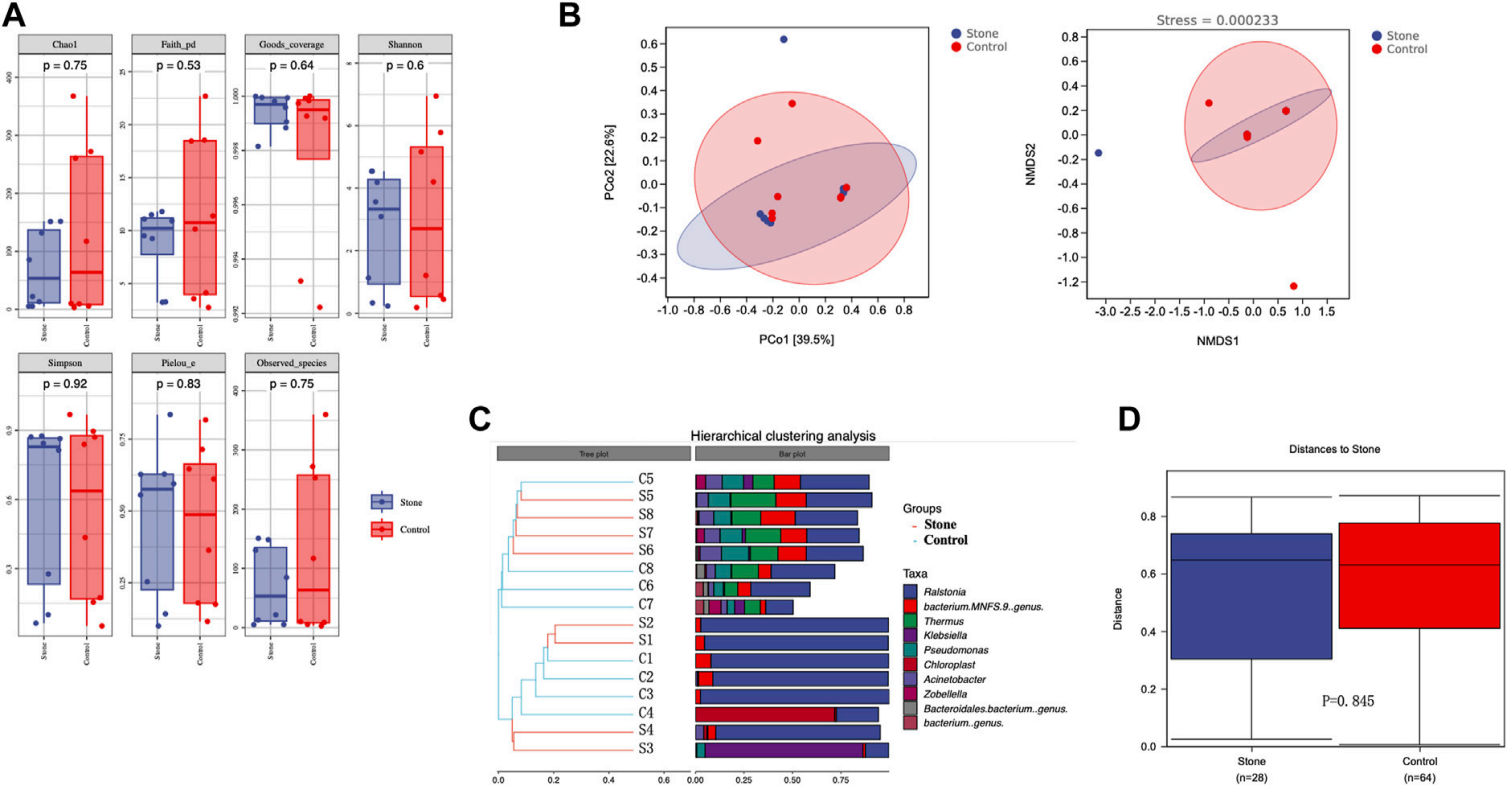
Richness and diversity of the microbiota in the pelvic urine samples from the stone and non-stone sides. **(A)** Alpha diversity, also known as within-habitat diversity, was used to evaluate the species richness, diversity, and evenness. Chao1, Good’s coverage, Simpson, Pielou’s evenness, Faith’s PD, Shannon, and Observed species indices indicate the alpha diversity of the pelvic urine samples on the stone and non-stone sides. **(B)** PCoA scatter and NMDS scatter plots were constructed to explore the beta diversity of the pelvic urine microbiota on both stone and non-stone sides using Jaccard analysis. **(C)** Hierarchical clustering analysis. **(D)** Difference between groups. PD, phylogenetic diversity; PCoA, principal coordinate analysis; NMDS, nonmetric multi-dimensional scaling.

Alpha diversity is an indicator of the microbiota’s richness, diversity, and evenness and is also known as within-habitat diversity. The alpha diversity indices, which include Chao1, Simpson, Shannon, Good’s coverage, Pielou’s evenness, Faith’s PD, and Observed species, showed no significant differences in within-habitat diversity using the Kruskal–Wallis test (Figure 3A). Beta diversity refers to the difference in species composition or the rate of species replacement along an environmental gradient between different communities and is also known as between-habitat diversity. Regarding beta diversity, the PCoA and NMDS showed no obvious differences in the microbiota between the stone and non-stone sides based on the Bray–Curtis distance (Figure 3B). Since no significant differences were found between the two groups, we deeply explored each sample to determine the common composition. Hierarchical clustering analysis revealed clustering between the samples based on their similarity. Ralstonia was the predominant taxon in every clustering, irrespective of the sides (Figure 3C). PCoA and NMDS are only exploratory analysis methods rather than statistical test techniques. Therefore, the distribution patterns were verified, and Adonis analysis was used to confirm the existence of no significant differences between the two groups (*p* = 0.845) (Figure 3D).

### 3.4 Bacteria distributions among groups

**FIGURE 4:**
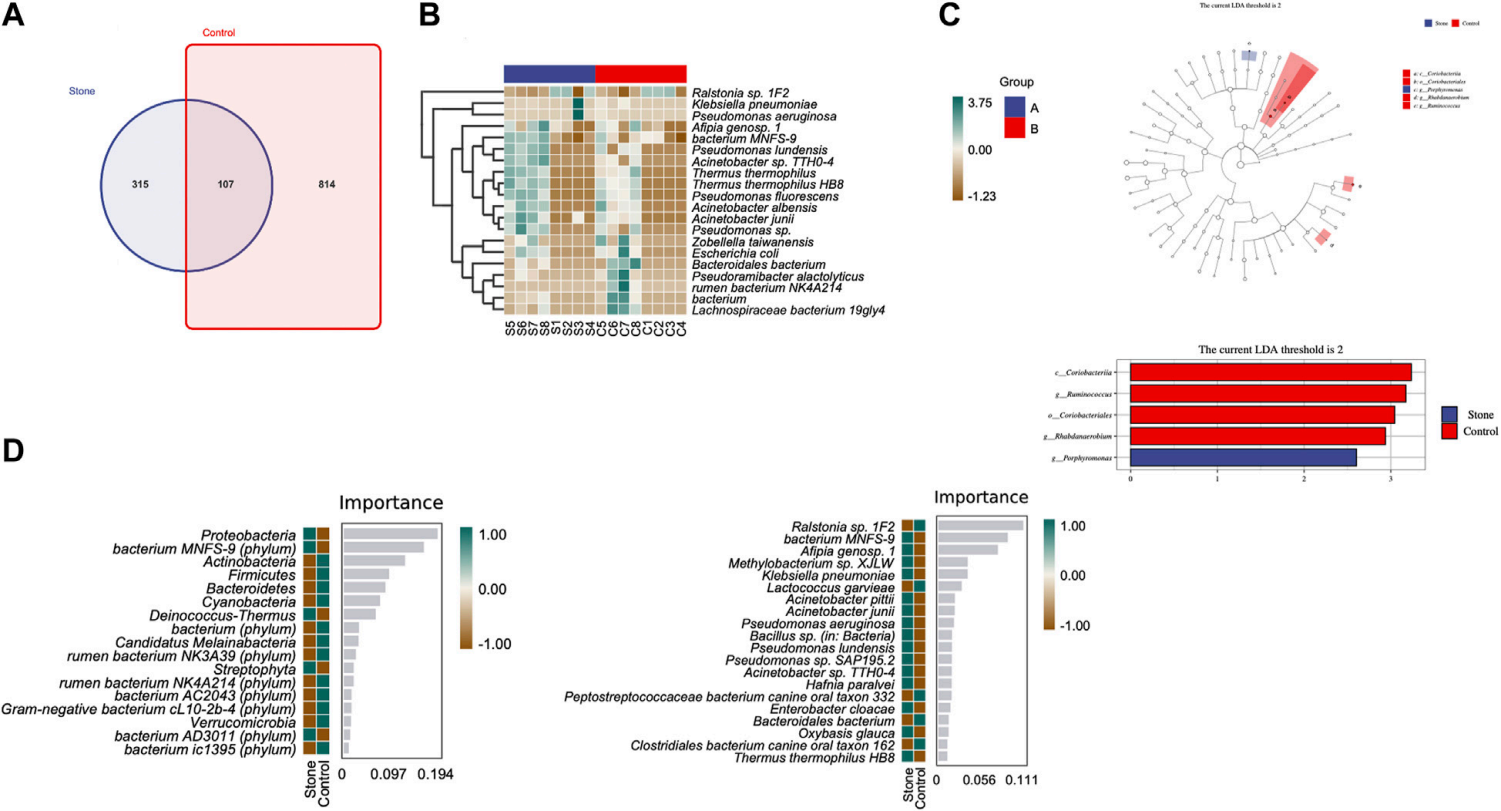
Differential abundance of microbiota between the pelvis urine samples from the stone and non-stone sides. **(A)** The Venn diagram indicates that the pelvic urine samples from the stone and non-stone sides share 107 common bacteria. **(B)** Heatmap of microbiota in the pelvis urine of each sample. **(C)** The cladogram represents the classification hierarchy relationship of the main classification units from phylum to species. The size of the node represents the abundance of the unit; hollow and solid nodes indicate no significant differences and significant differences, respectively. **(D)** Random forests. The bar chart shows the top 20 significantly different species/phylum between the stone and non-stone sides. Heatmap demonstrated the abundance of the top 20 bacteria in every pelvis urine sample.

The Venn diagram indicated that the stone and non-stone sides shared 107 ASVs in common: 315 and 814 on the stone and non-stone sides, respectively (Figure 4A). A heatmap was used for species composition analysis to further explore differences at the species level. Based on the screening of bacteria (*p* < 0.05), we identified the top 20 abundant bacteria at the species level. Among the 20 bacteria, Baterioidales, *Pseudoramibacter alactolyticus,* and rumen bacterium NK4A214 were the most stably enriched in pelvis urine samples from the stone sides (Figure 4B). We also applied LEfSe to determine the different abundant taxa on the stone and non-stone sides. LEfSe identified five discriminative features (LDA score ≥2.0) between the two groups. At the phylum level, the urobiomes on the non-stone sides were enriched with Coriobacteria. However, at the species level, the taxa that differentiated the two groups were Porphyromonas on the stone side and Ruminococcus and Rhabdanaerobium on the non-stone sides (Figure 4C). Therefore, to specifically distinguish the stone side samples from that of the non-stone sides, a random forest classifier was constructed. There were 17 bacteria at the phyla level to construct the classification models with an accuracy of 0.625, whereas 20 species were selected as potential markers to distinguish the stone sides from non-stone sides with an accuracy of 0.5 (Figure 4D), There were already some studies focused on the urinary microbiota of CaOx stones, we have presented the microbiota they found in Table 2. Compared with the microbiota previously found, there were many common bacteria, of which Proteobacteria, Actinobacteria, Hafnia, *Klebsiella* and *Enterobacter cloacae* were repeatedly enriched in bladder urine of CaOx stone patients or pelvis urine of stone sides, while Firmicute, Bacteroidetes and Lactococcus were repeatedly enriched in bladder urine of healthy volunteers of pelvis urine of non-stone sides.

**TABLE 2:** The urinary microbiota revealed previously through 16S rRNA gene sequencing.

Year	Author	Method	Materials	Stones	Different bacteria
2019	RyanA.Dornbier	16SrRNAgene sequencing	Bladder urine and Calcium based stones	Calcium-based stones	*Staphylococcus* epidermidis
					*Enterobacter cloacae*
					*Escherichia coli*
					*Lactobacillus* gasseri
					*Pseudomonas*
					Veillonella
					*Streptococcus*
					Corynebacterium
					*Haemophilus*
					Bifidobacterium
					*Proteus*
					Anaerobic cocci
2020	JingXie	16SrRNAgene sequencing	Healthy bladder urine Stone bladder urine Stone pelvis urine	CaOx &Caphos	Bacteroidetes
					Proteobacteria
					Firmicutes
					Faecalibacterium
					*Lactobacillus*
					Anoxybacillus
					*Fusobacterium*
					Prevotella
					*Acinetobacter*
					Anoxybacillus
2020	Fengping Liu	16SrRNAgene sequencing	Stone pelvis urine Non-stone pelvis urine	Calcium stones	Acetobacteraceae
					Acidovorax
					Aerococcus
					Agrococcus
					Arthrobacter
					Brevibacterium
					Brevundimonas
					*Clostridium*
					Curtobacterium
					Elizabethkingia
					Herbaspirillum
					Janthinobacterium
					Jeotgalicoccus
					Kocuria
					Lactococcus
					Memnoniella
					Microbacterium
					*Moraxella*
					Myrmecridium
					Nigrospora
					Paenibacillus
					Paenisporosarcina
					Pseudoclavibacter
					*Pseudomonas*
					Psychrobacter
					Rhodococcus
					Roseomonas
					Saccharopolyspora
					Sphingomonas
					*Staphylococcus*
					*Streptococcus*
					Bifidobacterium
					*Acinetobacter*
					Propionibacterium
					Delftia
					Pontibacter
					Corynebacterium
					*Lactobacillus*
2022	Yuanyuan Yang	16SrRNAgene sequencing	Pelvis urine of stone side Pelvis urine of non-stone side	CaOx stones	Thermus thermophilus
					*Acinetobacter*
					Pseudomonaslundensis
					*Enterobacter* asburiae
					*Enterobacter cloacae*
					Herbaspirillum huttiense
					Acidovorax
					Hafnia paralvei
					Chryseobacterium
					Akkermansia glycaniphila
					*Clostridium*
					Ruminococcus
					Rhabdanaerobium
					Oscillibacter valericigenes
					Neglecta timonensis
					*Klebsiella* quasipneumoniae
					Ruthenibacterium lactatiformans
					Slackia faecicanis

### 3.5 Potential functional pathways associated with CaOx stones

**FIGURE 5:**
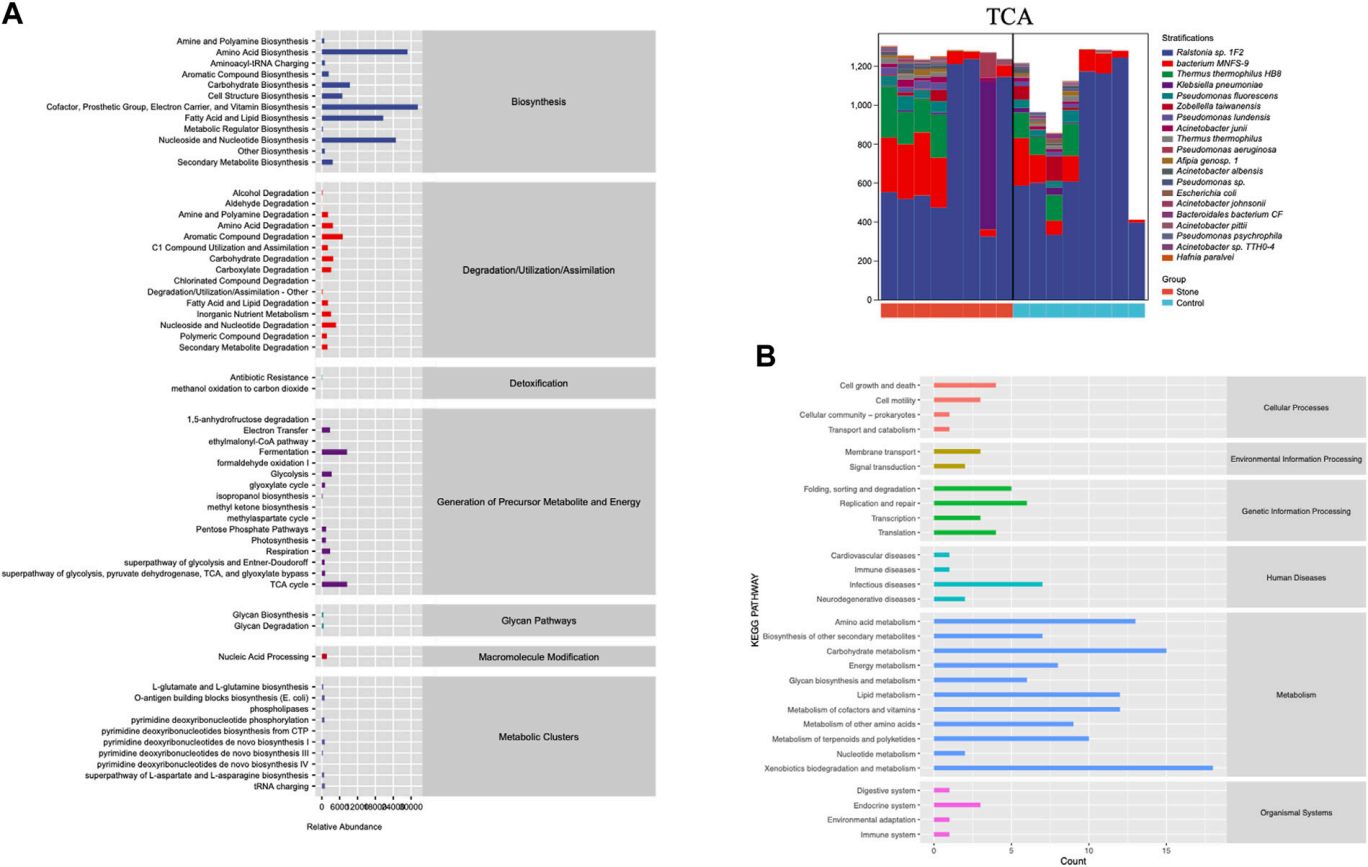
The function of the significantly different microbiota. **(A)** MetaCyc analysis of different microbiota from the pelvis urine samples of both the stone and non-stone sides. **(B)** KEGG analysis of significantly different microbiota. KEGG, Kyoto Encyclopedia for Genes and Genomes.

PICRUSt2 was applied to predict the potential function of the pelvic urine microbiota. It was just a prediction of the possibility, it did not draw a conclusion of specific function. This prediction could provide a general understanding of the possible functions and shed light on the subsequent shotgun metagenomic sequencing. We conducted the KEGG and MetaCyc analysis to explore the biological functions of the bacteria that were significantly different between the stone and non-stone sides (Figure 5). MetaCyc analysis showed that the most enriched pathways were related to biosynthesis (amino acid biosynthesis, cofactor, prosthetic, group, electron carrier, and vitamin biosynthesis), degradation/utilization/assimilation (aromatic compound degradation), detoxification (antibiotic resistance), and the generation of precursor metabolites and energy (fermentation and tricarboxylic acid (TCA) cycle). The top 20 bacteria at the species level involved in the TCA cycle are shown in Figure 4B. *Ralstonia* sp.1F2 was the dominant bacterium in each pelvic urine sample. The KEGG analysis indicated that the pathways focused on cellular processes (cell growth and death), environmental information processing (membrane transport), genetic information processing (replication and repair), human diseases (immune and infectious diseases), and organismal systems (endocrine system).

## 4 Discussion

CaOx stone is a common disease with high morbidity and recurrence rates. Genomic studies have shown that CaOx stones are associated with oxidative stress, inflammation, immunity, and complement activation pathways (Okada et al., 2009). With the rapid development of 16S rRNA gene sequencing and expanded quantitative urine culture, Dornbier et al. (2020) found that urine was not sterile but colonized by specialized microbiota related to many urological diseases. Previously, there were just a few studies focusing on urine microbiota related to metabolic stones; however, more and more studies demonstrated that most bacteria are isolated from metabolic stones, such as CaOx stones, rather than infectious stones (Tavichakorntrakool et al., 2012; Chen et al., 2019). Additionally, the difference in urine microbial composition between healthy people and patients with stones indicated that bacterial colonization affected stone formation (Liu et al., 2020a; Liu et al., 2020b). Clinically, the occurrence of CaOx stones is frequently unilateral (Durbin et al., 2012); therefore, there must be some factors affecting the local environment rather than systemic factors (Xie et al., 2020). To verify our hypothesis, we collected the pelvic urine samples of patients with unilateral CaOx stones from the stone and non-stone sides to set a self-control. Furthermore, we aimed to identify the differences in the colonized microbiota between the healthy and stone sides of the same patient and further explore how this microbiota affects the formation of CaOx stones.

Although Based on the alpha and beta diversity results, no significant difference was found in the overall microbiota communities between the stone and non-stone sides, some specific taxa still showed different expressions between the two sides. The resemblance of the microbiota between the stone and non-stone sides was consistent with a previous study (Liu et al., 2020b). Therefore, we assumed the existence of several possibilities. First, the diversity between the stone and non-stone sides was similar when they were from the same patient. Second, contamination of the bladder during the collection process resulted in a mixture of microbiota between the stone and non-stone sides, although we attempted to minimize retrograde bladder contamination. Third, we could not exclude the possibility that the patients might have a trend of bilateral CaOx stones that have not yet formed or that they had bilateral CaOx stones in the past, but the stone was tiny and was eliminated in the urine. Our previous study found that the overall microbiota differed, which might be because of the limited sample size (Kachroo et al., 2021). Therefore, animal model experiments and follow-up studies with larger sample sizes should be conducted to verify these possibilities.

The microbime we found were not absolutely similar to previous stuies, Dorbier et al. found that *Staphylococcus*, Enterbacter cloacae, *Escherichia coli*, Corynebacterium and *Lactobacillus* enriched in bladder urine with calcium-based stones, while Liu F et al. and Xie J et al. added to these bacterial communities with additional identification of Proteobacteria, Firmicute and *Acinetobacter* (Liu et al., 2020b; Dornbier et al., 2020; Xie et al., 2020). In our research, we sequenced the pelvis urine of both two sides of the same unilateral CaOx stone patient, the abundance profiles indicated that the predominant microbiota was relatively similar between the stone and non-stone sides at the phylum and species levels. However, there were still many different bacteria between the 2 groups, previous studies and our research shared many common microbiota, we have listed the most common ones along with their characteristics in Table 3, of which Proteobacteria, Actinobacteria, Hafnia, *Klebsiella* and *E. cloacae* were repeatedly enriched in bladder urine of CaOx stone patients or pelvis urine of stone sides, while Firmicute, Bacteroidetes and Lactococcus were repeatedly enriched in bladder urine of healthy volunteers of pelvis urine of non-stone sides. Despite the common dominant bacterial communities, our current study adds to these bacterial communities with additional identification of Deinococcus-Thermus, Coriobacteriia, Porphyromonas and Ralstonia. Proteobacteria, has been observed in various human body sites, including the oral cavity, skin, vaginal tract, and urinary tract (Costello et al., 2009; Caporaso et al., 2011; Human Microbiome Project Consortium, 2012). Proteobacteria are important for maintaining a balanced microbial community, and an increased prevalence of Proteobacteria is a potential diagnostic criterion for disease. For example, Proteobacteria can cause metabolic diseases such as type 2 diabetes mellitus by affecting glucose homeostasis (Qin et al., 2012). The immune system strictly regulates its responses to maintain a symbiotic relationship with commensal bacteria, and many studies have demonstrated that Proteobacteria can destroy the immune balance and cause inflammation (Walujkar et al., 2014). The uncontrolled expansion of Proteobacteria further facilitates inflammation and invasion. The function of Firmicutes is similar to that of Proteobacteria, and the presence of Firmicutes disrupt the balance of immunity and cause inflammation (Verdam et al., 2013). Deinococcus-Thermus is mostly found in the natural environment and has been rarely reported in human infections, except by Yi Zhang et al., who found that it might be involved in hepatitis B formation (Zhang et al., 2019). At the species level, the predominant bacteria included *Ralstonia* sp., *Pseudomonas*, and *Acinetobacter*, of which *Pseudomonas* and *Acinetobacter* have also been previously reported as the dominant species in pelvis urine (Liu et al., 2020b). *Acinetobacter* and *Pseudomonas* are also important opportunistic pathogens that cause UTIs (Xie et al., 2020). Furthermore, *Pseudomonas* was reportedly dominant in stone homogenate (Dornbier et al., 2020), while *Ralstonia* sp. is mainly found in the lungs and can causes inflammation and fibrosis (Coenye et al., 2003).

**TABLE 3:** The bacteria common in the current study and previous research findings in renal pelvis urine.

Taxa	Gram staining	Group	Characteristic description
Proteobacteria	Negative	Stone	Type 2 diabetes Inflammation disease
Actinobacteria	Negative	Stone	Opportunistic pathogens for UTIs
Hafnia	Negative	Stone	Related to Inflammation and Fibrosis
*Klebsiella*	Negative	Stone	Bacterial hepatic abscess
*Enterobacter cloacae*	Negative	Stone	Catalase positive
Firmicute	Positive	Control	Balance of immunity
Bacteroidete	Negative	Control	Opportunistic infection
Porphyromonas	Negative	Stone	Immune system maturation
Lactococcus	Positive	Control	Gut colonized bacteria
Coriobacteriia	Positive	Control	Balance of immunity
Ralstonia	Negative	Control	Hospital infection

Through paired compositions and LEfSe at the phylum level, the urobiome of the non-stone sides was enriched with Coriobacteriia. However, at the species level, Porphyromonas and Ruminococcus on the stone and non-stone sides, respectively, were the taxa that differentiated the two groups. Coriobacteriia is a gut microflora, and Ibrahim Yusufu found that Coriobacteriia is associated with inflammation by disrupting the balance of immunity (Yusufu et al., 2021). Porphyromonas was one of the top 10 anaerobic taxa in the microbiome in healthy patients, and it appeared that Porphyromonas was described in various other body sites, including the lungs, skin, stomach, urinary tract, and vagina (Hillier et al., 1993; Hoen et al., 2015; Lim et al., 2016; Ramadan et al., 2019). Pattaroni et al. found this to be a key determinant of immune system maturation (Pattaroni et al., 2018). Although many studies on Porphyromonas have been conducted, they were majorly associated with pulmonary diseases. Ruminococcus has been identified as a strict anaerobe in the gut of healthy individuals (Crost et al., 2023). Grabinger et al. (2019) demonstrated that Ruminococcus can decrease the severity of inflammation in mouse models of colitis. Ahn et al. (2022) found that Ruminococcus can modulate the immune system by enhancing regulatory T-cell counts and SCFA production. Bioinformatics analysis showed that the significantly different bacteria were mainly enriched in the immune system and metabolic pathways. Many studies have verified the association between SCFAs, microbiota, and CaOx stone disease (Liu et al., 2020c). Notably, SCFAs are the main metabolites of the microbiota that play an important role in immunomodulation and can also regulate oxidative stress to prevent acute and chronic kidney injury, which are the two main pathways leading to stone formation (Li et al., 2017; Duscha et al., 2020). Furthermore, the microbiome has been proven to play a role in modulating metabolism and immunity in many diseases (Michaudel and Sokol, 2020).

Immunometabolism was introduced in 2002 to explore the interactions between immune cells and metabolism (Frauwirth et al., 2002). Our bioinformatics analysis revealed that the TCA cycle was the dominant pathway involved in metabolism, which is consistent with a previous study. Additionally, Bantug et al. (2018) indicated that TCA is one of the main metabolic pathways of immunometabolism. SCFA is one of the metabolic producers of bacteria that impact the metabolism of immune cells (Goodpaster and Sparks, 2017). Xijin et al. demonstrated that SCFAs inhibit CaOx crystal formation by modulating the functions of microbiota-dependent macrophages (Jin et al., 2021). Therefore, we hypothesized that the metabolic product of the kidney-colonized microbiome could modulate the immune environment, affecting the formation of kidney stones. Moreover, 17 species and phyla were selected as optimal markers to construct a random forest model that could distinguish the stone side from the non-stone side, with an accuracy of 62.5%.

Based on previous studies, we know that bacteria generally not only cause diseases alone but also form microbial communities that affect local metabolism, alter the immune microenvironment, and affect the formation of kidney stones. Our study had some limitations. First, our study had only 8 pairs of matched samples which was not sufficiently large, and it was not a multicenter study, which limited the subgroup analysis. Second, this was only a descriptive study, and the specific mechanism needs to be verified in subsequent animal experiments. However, to compensate for the insufficient sample size, we referenced many other studies and presented them in Table 2. We compared these results and identified the most frequently occurring bacteria in urine. In addition, in future prospective studies, we will explore the feasibility of using microbiota as biomarkers of stone formation. We will elucidate the crosstalk among the microbiota, immunometabolism, and CaOx stones to provide insight into the medical treatment and prevention of CaOx stones.

In conclusion, our study pioneered the collection of pelvis urine samples from the non-stone and stone sides of the same patients with CaOx stones to set self-control and conducted 16S rRNA gene sequencing. Although no statistically significant differences were found in the overall microbiome of the bilateral pelvis urine samples, many biologically distinct taxa were observed, including many bacteria found in previous studies, like Proteobacteria, Actinobacteria, firmicute and *E. cloacae*. What’s more, we also found some bacteria that have not been reported, like Ralstonia, Deinococcus-Thermus, Coriobacteriia and Porphyromonas. Prediction of the function of these different bacteria showed they might be associated with metabolic and immune responses. In addition, the pelvis urine microbiome have a potential to be liquid biomarkers for CaOx stones. Our study, for the first time, compared the differences in renal pelvis urine between the healthy side and the side with stones in the same patient, while excluding the influence of systemic factors. However, our study was just a preliminary research with a tiny sample size, it was just a descriptive study to present the microbiome composition of the stone sides and control sides of the same patients with unilateral CaOx stones. More patients will be recruited in the future and further experiments are required to explore the crosstalk among urinary microbiota, immunometabolism, and CaOx stones.

## Figures and Tables

**Table:** 

**CaOx**	Calcium Oxalate
**SCFAs**	Short-chain fatty acids
**UTI**	Urinary tract infection

## Data Availability

The data presented in the study are deposited in the SRA repository, accession number PRJNA970228.

## Abbreviations

ASV, amplicon sequence variant; CaOx, Calcium Oxalate stone; KEGG, Kyoto Encyclopedia of Genes and Genomes; OUT, operational taxonomic units.
