# Supplementary figures and images for: The genetics of urinary microbiome, an exploration of the trigger in calcium oxalate stone
Source: Front Genet. 2023 Oct 3;14:1260278. doi: 10.3389/fgene.2023.1260278 (PMC10579592; doi:10.3389/fgene.2023.1260278)

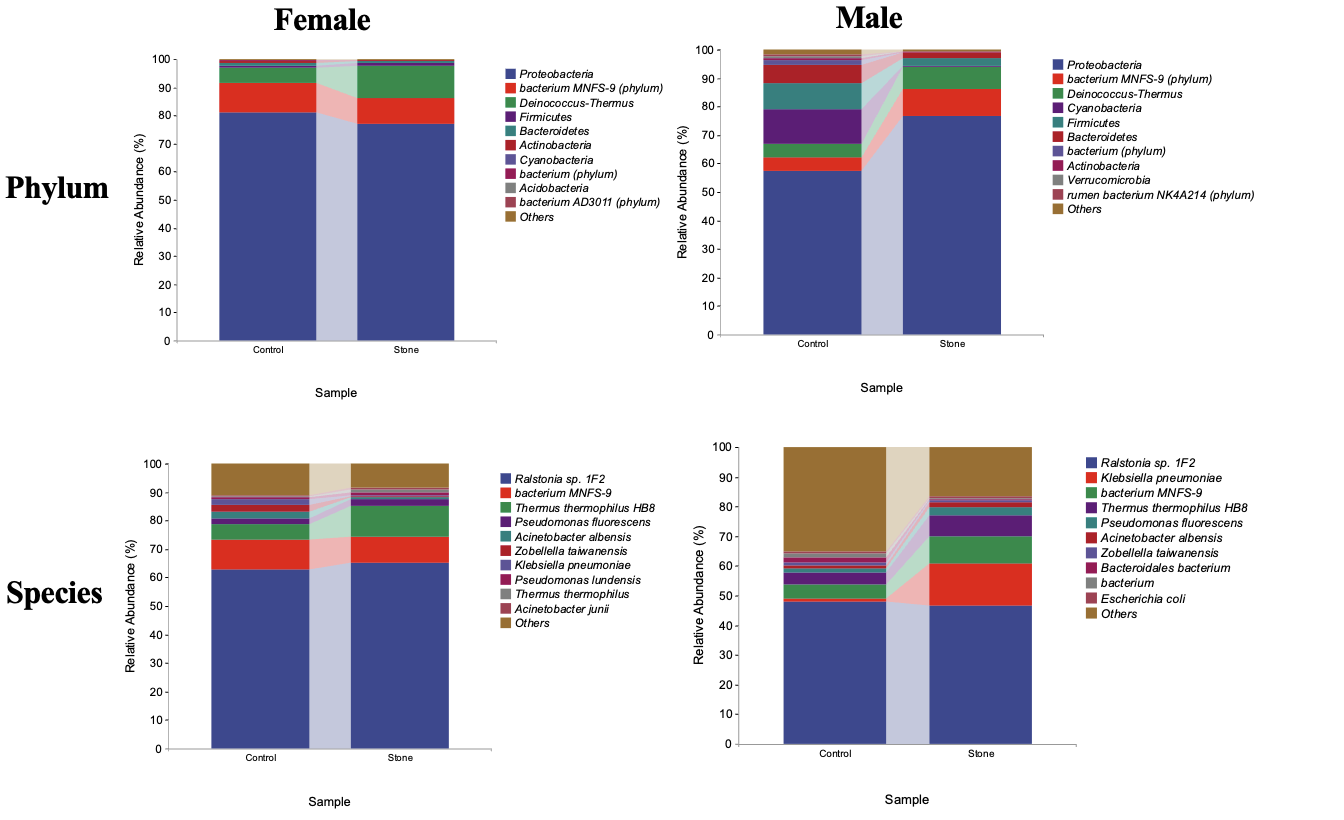

Supplement: Supplementary file 2 [file Image1.png]
